# *catena*-Poly[[bis­(pyridine-3-carb­oxy­lic acid-κ*N*)mercury(II)]-di-μ-chlorido]

**DOI:** 10.1107/S1600536812012986

**Published:** 2012-03-31

**Authors:** Sadif A. Shirvan, Sara Haydari Dezfuli

**Affiliations:** a Department of Chemistry, Omidieh Branch, Islamic Azad University, Omidieh, Iran

## Abstract

In the title compound, [HgCl_2_(C_6_H_5_NO_2_)_2_]_*n*_, the Hg^II^ cation is located on an inversion center and is six-coordinated in a distorted octa­hedral geometry by two N atoms from two pyridine-3-carb­oxy­lic acid mol­ecules and four bridging Cl^−^ anions. The bridging function of the Cl^−^ anions leads to polymeric chains running along the *a* axis. One Hg—Cl bond is much longer than the other. In the crystal, O—H⋯O and weak C—H⋯Cl hydrogen bonds are observed.

## Related literature

For related structures, see: Lu & Kohler (2002 ▶); Liang & Li (2005 ▶); Zhang *et al.* (1996 ▶); Ghazzali *et al.* (2007 ▶); Lin *et al.* (1998 ▶); Cotton *et al.* (1991 ▶).
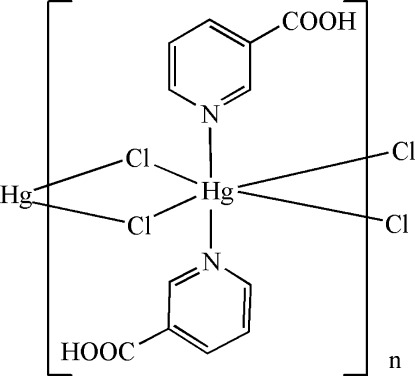


## Experimental

### 

#### Crystal data

[HgCl_2_(C_6_H_5_NO_2_)_2_]
*M* *_r_* = 517.71
Triclinic, 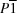

*a* = 3.8298 (5) Å
*b* = 6.5626 (9) Å
*c* = 14.5831 (18) Å
α = 98.001 (10)°
β = 95.315 (11)°
γ = 92.963 (11)°
*V* = 360.62 (8) Å^3^
*Z* = 1
Mo *K*α radiation
μ = 11.06 mm^−1^
*T* = 298 K
0.20 × 0.10 × 0.05 mm

#### Data collection

Bruker SMART 1000 CCD area-detector diffractometer
Absorption correction: multi-scan (*SADABS*; Bruker, 2001 ▶) *T*_min_ = 0.293, *T*_max_ = 0.523
4417 measured reflections
1917 independent reflections
1913 reflections with *I* > 2σ(*I*)
*R*_int_ = 0.078

#### Refinement

*R*[*F* ^2^ > 2σ(*F* ^2^)] = 0.032
*wR*(*F* ^2^) = 0.082
*S* = 0.83
1917 reflections
98 parameters
H-atom parameters constrained
Δρ_max_ = 1.02 e Å^−3^
Δρ_min_ = −1.22 e Å^−3^

### 

Data collection: *SMART* (Bruker, 2007 ▶); cell refinement: *SAINT* (Bruker, 2007 ▶); data reduction: *SAINT*; program(s) used to solve structure: *SHELXS97* (Sheldrick, 2008 ▶); program(s) used to refine structure: *SHELXL97* (Sheldrick, 2008 ▶); molecular graphics: *ORTEP-3 for Windows* (Farrugia, 1997 ▶); software used to prepare material for publication: *WinGX* (Farrugia, 1999 ▶).

## Supplementary Material

Crystal structure: contains datablock(s) I, global. DOI: 10.1107/S1600536812012986/xu5491sup1.cif

Structure factors: contains datablock(s) I. DOI: 10.1107/S1600536812012986/xu5491Isup2.hkl

Additional supplementary materials:  crystallographic information; 3D view; checkCIF report

## Figures and Tables

**Table 1:** Selected bond lengths (Å)

Hg1—Cl1	2.4608 (13)
Hg1—Cl1^i^	2.8790 (13)
Hg1—N1^ii^	2.519 (4)

Symmetry codes: (i) 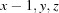; (ii) 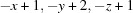.

**Table 2:** Hydrogen-bond geometry (Å, °)

*D*—H⋯*A*	*D*—H	H⋯*A*	*D*⋯*A*	*D*—H⋯*A*
O2—H2*A*⋯O1^iii^	0.82	1.80	2.618 (7)	171
C1—H1⋯Cl1^iv^	0.93	2.79	3.582 (6)	144
C6—H6⋯Cl1^ii^	0.93	2.78	3.454 (5)	130

Symmetry codes: (ii) 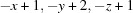; (iii) 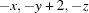; (iv) 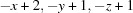.

**Fig. 1.**
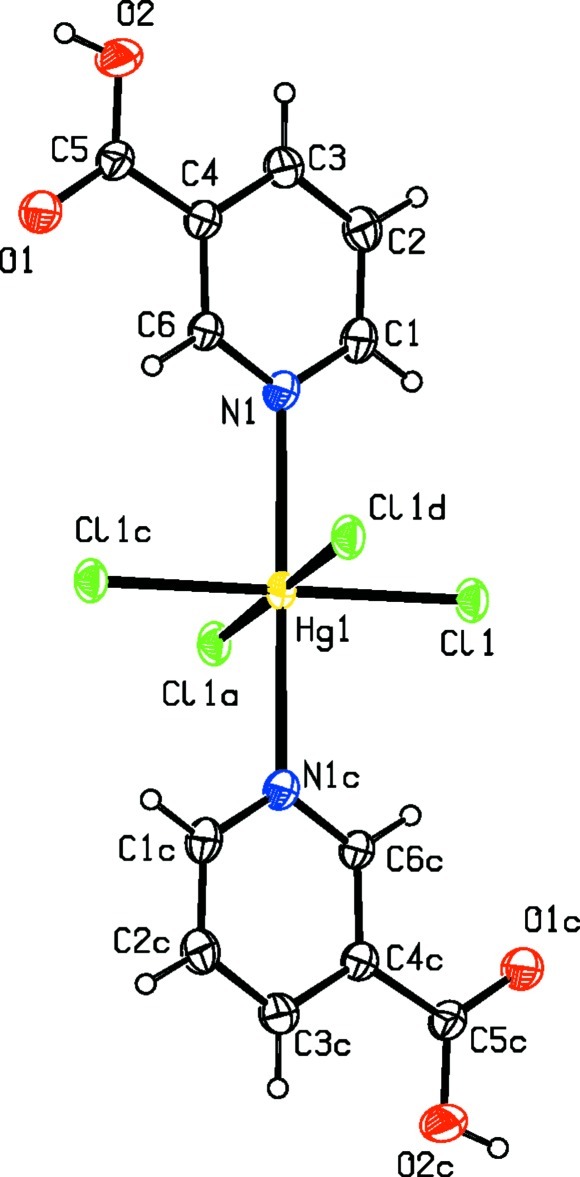
The molecular structure of the title molecule, with the atom-numbering scheme. Displacement ellipsoids are drawn at the 30% probability level. [Symmetry codes: (a) -1 + x,y,z; (c) 1 - x,2 - y,1 - z; (d) 2 - x,2 - y,1 - z].

**Fig. 2.**
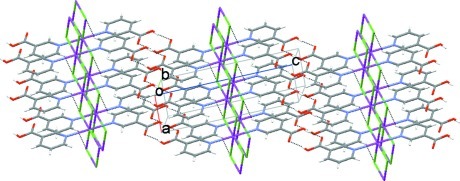
A packing diagram of the title complex. Hydrogen bonds are shown as dashed lines.

## supplementary crystallographic information

### Comment

3-Pyridine carboxylic acid (pyc), is a good ligand, and a few complexes with pyc have been prepared, such as that of cadmium (Lu & Kohler, 2002; Liang & Li, 2005; Zhang *et al.*, 1996) and zinc (Ghazzali *et al.*, 2007; Lin *et al.*, 1998; Cotton *et al.*, 1991). Here, we report the synthesis and structure of the title compound.

The asymmetric unit of the title compound, (Fig. 1), contains one half -molecule. The Hg^II^ atom is six-coordinated in a distorted octahedral configuration by two N atoms from two 3-pyridine carboxylic acid and four bridging Cl. The bridging function of the chloro atoms leads to a one-dimensional chain structure. The Hg—Cl and Hg—N bond lengths and angles are collected in Table 1.

In the crystal structure, intermolecular O—H···O and C—H···Cl hydrogen bonds (Table 2, Fig. 2) may stabilize the structure.

### Experimental

A solution of pyridine-3-carboxylic acid (0.25 g, 2.0 mmol) in methanol (20 ml) was added to a solution of HgCl_2_ (0.27 g, 1.0 mmol) in methanol (20 ml) and the resulting colorless solution was stirred for 15 min at room temperature. This solution was left to evaporate slowly at room temperature. After one week, colorless needle crystals of the title compound were isolated (yield 0.41 g, 79.2%).

### Refinement

H atoms were positioned geometrically, with C—H = 0.93 Å and constrained to ride on their parent atoms, with *U*_iso_(H) = 1.2*U*_eq_(C).

### Crystal data

**Table:**

[HgCl_2_(C_6_H_5_NO_2_)_2_]	*Z* = 1
*M**_r_* = 517.71	*F*(000) = 242
Triclinic, *P*1	*D*_x_ = 2.384 Mg m^−^^3^
Hall symbol: -P 1	Mo *K*α radiation, λ = 0.71073 Å
*a* = 3.8298 (5) Å	Cell parameters from 1010 reflections
*b* = 6.5626 (9) Å	θ = 2.8–29.2°
*c* = 14.5831 (18) Å	µ = 11.06 mm^−^^1^
α = 98.001 (10)°	*T* = 298 K
β = 95.315 (11)°	Needle, colorless
γ = 92.963 (11)°	0.20 × 0.10 × 0.05 mm
*V* = 360.62 (8) Å^3^	

### Data collection

**Table:**

Bruker SMART 1000 CCD area-detector diffractometer	1917 independent reflections
Radiation source: fine-focus sealed tube	1913 reflections with *I* > 2σ(*I*)
Graphite monochromator	*R*_int_ = 0.078
φ and ω scans	θ_max_ = 29.2°, θ_min_ = 2.8°
Absorption correction: multi-scan (*SADABS*; Bruker, 2001)'	*h* = −4→5
*T*_min_ = 0.293, *T*_max_ = 0.523	*k* = −8→8
4417 measured reflections	*l* = −19→19

### Refinement

**Table:**

Refinement on *F*^2^	Primary atom site location: structure-invariant direct methods
Least-squares matrix: full	Secondary atom site location: difference Fourier map
*R*[*F*^2^ > 2σ(*F*^2^)] = 0.032	Hydrogen site location: inferred from neighbouring sites
*wR*(*F*^2^) = 0.082	H-atom parameters constrained
*S* = 0.83	*w* = 1/[σ^2^(*F*_o_^2^) + (0.0827*P*)^2^] where *P* = (*F*_o_^2^ + 2*F*_c_^2^)/3
1917 reflections	(Δ/σ)_max_ = 0.015
98 parameters	Δρ_max_ = 1.02 e Å^−^^3^
0 restraints	Δρ_min_ = −1.22 e Å^−^^3^

### Special details

**Table:**

Geometry. All e.s.d.'s (except the e.s.d. in the dihedral angle between two l.s. planes) are estimated using the full covariance matrix. The cell e.s.d.'s are taken into account individually in the estimation of e.s.d.'s in distances, angles and torsion angles; correlations between e.s.d.'s in cell parameters are only used when they are defined by crystal symmetry. An approximate (isotropic) treatment of cell e.s.d.'s is used for estimating e.s.d.'s involving l.s. planes.
Refinement. Refinement of *F*^2^ against ALL reflections. The weighted *R*-factor *wR* and goodness of fit *S* are based on *F*^2^, conventional *R*-factors *R* are based on *F*, with *F* set to zero for negative *F*^2^. The threshold expression of *F*^2^ > σ(*F*^2^) is used only for calculating *R*-factors(gt) *etc*. and is not relevant to the choice of reflections for refinement. *R*-factors based on *F*^2^ are statistically about twice as large as those based on *F*, and *R*- factors based on ALL data will be even larger.

### Fractional atomic coordinates and isotropic or equivalent isotropic displacement parameters (Å^2^)

**Table:**

	*x*	*y*	*z*	*U*_iso_*/*U*_eq_	
C1	0.5737 (16)	0.5901 (8)	0.3289 (4)	0.0441 (10)	
H1	0.6553	0.5307	0.3805	0.053*	
C2	0.5746 (17)	0.4781 (8)	0.2408 (4)	0.0471 (11)	
H2	0.6561	0.3463	0.2338	0.057*	
C3	0.4531 (16)	0.5643 (8)	0.1635 (4)	0.0444 (10)	
H3	0.4528	0.4926	0.1038	0.053*	
C4	0.3308 (14)	0.7624 (7)	0.1775 (3)	0.0376 (8)	
C5	0.1917 (15)	0.8656 (8)	0.0996 (3)	0.0419 (9)	
C6	0.3410 (14)	0.8644 (7)	0.2680 (3)	0.0385 (8)	
H6	0.2618	0.9966	0.2770	0.046*	
N1	0.4590 (13)	0.7817 (7)	0.3425 (3)	0.0410 (8)	
O1	0.0639 (17)	1.0378 (8)	0.1165 (3)	0.0595 (12)	
O2	0.2082 (19)	0.7727 (9)	0.0170 (3)	0.0648 (14)	
H2A	0.1115	0.8387	−0.0208	0.097*	
Cl1	0.9257 (3)	0.77277 (19)	0.56211 (9)	0.0418 (2)	
Hg1	0.5000	1.0000	0.5000	0.03700 (10)	

### Atomic displacement parameters (Å^2^)

**Table:**

	*U*^11^	*U*^22^	*U*^33^	*U*^12^	*U*^13^	*U*^23^
C1	0.047 (3)	0.041 (2)	0.047 (2)	0.0095 (19)	−0.0002 (19)	0.0179 (18)
C2	0.055 (3)	0.035 (2)	0.053 (3)	0.0128 (19)	0.002 (2)	0.0103 (18)
C3	0.051 (3)	0.039 (2)	0.044 (2)	0.0099 (19)	0.0038 (19)	0.0065 (16)
C4	0.040 (2)	0.0376 (19)	0.0365 (19)	0.0052 (16)	0.0025 (15)	0.0083 (15)
C5	0.048 (3)	0.042 (2)	0.037 (2)	0.0091 (19)	−0.0016 (17)	0.0081 (16)
C6	0.046 (2)	0.0380 (19)	0.0340 (19)	0.0129 (17)	0.0010 (16)	0.0103 (15)
N1	0.047 (2)	0.0413 (19)	0.0361 (17)	0.0116 (17)	−0.0005 (15)	0.0119 (14)
O1	0.085 (4)	0.054 (2)	0.0421 (19)	0.032 (2)	0.000 (2)	0.0115 (16)
O2	0.098 (4)	0.064 (3)	0.0336 (18)	0.033 (3)	−0.001 (2)	0.0055 (16)
Cl1	0.0408 (5)	0.0432 (5)	0.0452 (5)	0.0145 (4)	0.0031 (4)	0.0164 (4)
Hg1	0.03475 (14)	0.04550 (15)	0.03275 (13)	0.01720 (9)	0.00034 (8)	0.00937 (8)

### Geometric parameters (Å, º)

**Table:**

C1—N1	1.348 (7)	C5—O1	1.257 (7)
C1—C2	1.390 (8)	C5—O2	1.281 (7)
C1—H1	0.9300	C6—N1	1.334 (6)
C2—C3	1.384 (8)	C6—H6	0.9300
C2—H2	0.9300	N1—Hg1	2.519 (4)
C3—C4	1.399 (7)	O2—H2A	0.8200
C3—H3	0.9300	Hg1—Cl1	2.4608 (13)
C4—C6	1.390 (6)	Hg1—Cl1^i^	2.8790 (13)
C4—C5	1.474 (7)	Hg1—N1^ii^	2.519 (4)
			
N1—C1—C2	122.4 (5)	C6—N1—Hg1	118.5 (3)
N1—C1—H1	118.8	C1—N1—Hg1	123.2 (3)
C2—C1—H1	118.8	C5—O2—H2A	109.5
C3—C2—C1	119.4 (5)	Hg1—Cl1—Hg1^iii^	91.31 (4)
C3—C2—H2	120.3	Cl1—Hg1—Cl1^ii^	180.000 (1)
C1—C2—H2	120.3	Cl1—Hg1—N1^ii^	89.75 (11)
C2—C3—C4	118.2 (5)	Cl1^ii^—Hg1—N1^ii^	90.25 (11)
C2—C3—H3	120.9	Cl1—Hg1—N1	90.25 (11)
C4—C3—H3	120.9	Cl1^ii^—Hg1—N1	89.75 (11)
C3—C4—C6	118.7 (5)	N1^ii^—Hg1—N1	180.000 (1)
C3—C4—C5	122.1 (4)	Cl1—Hg1—Cl1^i^	91.31 (4)
C6—C4—C5	119.2 (5)	Cl1^ii^—Hg1—Cl1^i^	88.69 (4)
O1—C5—O2	123.2 (5)	N1^ii^—Hg1—Cl1^i^	85.85 (11)
O1—C5—C4	119.4 (5)	N1—Hg1—Cl1^i^	94.15 (11)
O2—C5—C4	117.4 (5)	Cl1—Hg1—Cl1^iv^	88.69 (4)
N1—C6—C4	123.1 (5)	Cl1^ii^—Hg1—Cl1^iv^	91.31 (4)
N1—C6—H6	118.4	N1^ii^—Hg1—Cl1^iv^	94.15 (11)
C4—C6—H6	118.5	N1—Hg1—Cl1^iv^	85.85 (11)
C6—N1—C1	118.1 (5)	Cl1^i^—Hg1—Cl1^iv^	180.0
			
N1—C1—C2—C3	−0.2 (9)	Hg1^iii^—Cl1—Hg1—N1^ii^	−94.16 (11)
C1—C2—C3—C4	−0.5 (9)	Hg1^iii^—Cl1—Hg1—N1	85.84 (11)
C2—C3—C4—C6	0.8 (8)	Hg1^iii^—Cl1—Hg1—Cl1^i^	180.0
C2—C3—C4—C5	−179.1 (5)	Hg1^iii^—Cl1—Hg1—Cl1^iv^	0.0
C3—C4—C5—O1	175.3 (6)	C6—N1—Hg1—Cl1	−159.3 (4)
C6—C4—C5—O1	−4.6 (8)	C1—N1—Hg1—Cl1	15.9 (4)
C3—C4—C5—O2	−4.6 (8)	C6—N1—Hg1—Cl1^ii^	20.7 (4)
C6—C4—C5—O2	175.4 (6)	C1—N1—Hg1—Cl1^ii^	−164.1 (4)
C3—C4—C6—N1	−0.7 (8)	C6—N1—Hg1—N1^ii^	−6 (100)
C5—C4—C6—N1	179.3 (5)	C1—N1—Hg1—N1^ii^	169 (100)
C4—C6—N1—C1	0.1 (8)	C6—N1—Hg1—Cl1^i^	109.4 (4)
C4—C6—N1—Hg1	175.5 (4)	C1—N1—Hg1—Cl1^i^	−75.4 (4)
C2—C1—N1—C6	0.4 (8)	C6—N1—Hg1—Cl1^iv^	−70.6 (4)
C2—C1—N1—Hg1	−174.8 (4)	C1—N1—Hg1—Cl1^iv^	104.6 (4)
Hg1^iii^—Cl1—Hg1—Cl1^ii^	73 (100)		

Symmetry codes: (i) *x*−1, *y*, *z*; (ii) −*x*+1, −*y*+2, −*z*+1; (iii) *x*+1, *y*, *z*; (iv) −*x*+2, −*y*+2, −*z*+1.

### Hydrogen-bond geometry (Å, º)

**Table:**

*D*—H···*A*	*D*—H	H···*A*	*D*···*A*	*D*—H···*A*
O2—H2*A*···O1^v^	0.82	1.80	2.618 (7)	171
C1—H1···Cl1^vi^	0.93	2.79	3.582 (6)	144
C6—H6···Cl1^ii^	0.93	2.78	3.454 (5)	130

Symmetry codes: (ii) −*x*+1, −*y*+2, −*z*+1; (v) −*x*, −*y*+2, −*z*; (vi) −*x*+2, −*y*+1, −*z*+1.
